# Medical Opinion and Movement

**Published:** 1906-12-22

**Authors:** 


### Medical Opinion and Movement.

Medical men are beginning to realise the importance of the present movement to check hospital abuse and are showing their earnestness in the matter by holding meetings in all parts of the country. At a meeting of the Marylebone Division of the British Medical Association, at which Dr. Lauriston Shaw presided, Sir R. Douglas Powell, in moving the following resolution—

That it is desirable that concerted action should be taken to check the large amount of gratuitous medical attendance granted to many sections of the community, inasmuch as it tends to diminish the thrift and self-respect of the public, lessens the esteem in which medical science is held, and has a detrimental effect upon the position of many members of the medical profession.

urged the development of provident dispensaries and sick-assurance societies as a means of lessening gratuitous medical services, and pointed out that the public should be educated to the necessity of contributing a fraction of their earnings during health for adequate medical treatment during sickness. He made the important suggestion that for efficient treatment of the lower middle class and artisans at their homes, a considerable staff of district nurses should be provided and that there should be depots established, where dressings and appliances could be obtained at a reasonable rate. The motion was seconded by Sir John Tweedy, who pointed out that the hospital system as at present administered tends to foster many of the evils which are found to attend indiscriminate almsgiving; he laid stress on the evils of overcrowding at hospital out-patients' departments as tending to hurried and perfunctory treatment and unfair competition with the practitioners of the neighbourhood. An interesting discussion followed, and the motion was carried, with one dissentient.

The first election of direct representatives of the General Medical Council held under the new regulations of the Privy Council establishing voting by ballot, presents several interesting features. The election on the present occasion had a special interest owing to the active part taken by the British Medical Association. Never before has there been so much discussion and publicity, nor, we may conclude, has there ever been so much organised influence brought to bear in an election of direct representatives. In such circumstances it was to be expected that the number of votes cast under the ballot system would show a marked increase on those given at any previous election. As a matter of fact, the number of voting papers returned was the smallest number ever given, with the exception of two elections. Less than fifty per cent. of the constituents took part in the voting. We take it that this result proves that the profession as a body are against organised effort on the part of a professional organisation to secure the return of their candidates to the exclusion of everyone else. If this view is correct it shows a wholesome state of opinion in the profession, and cannot fail to emphasise the importance which the profession attaches to the individual independence of its members. It is not a little remarkable, and is certainly unsatisfactory, that on the first occasion of a vote being taken by a regulated ballot, no less than 1,503 voting papers, or 12.61 per cent., were found to be invalid, as one or other of the prescribed conditions had not been fulfilled. Thus 615 electors failed to attach their signature to the identification envelope; 467 others failed to fasten the envelope and so forfeited their vote; 193 did not place the voting-paper inside the identification envelope, and so were disqualified; 35 papers were not marked with a cross; and 165 arrived too late to be included in the ballot. The election figures are disappointing. They fail to show any genuine increase of interest by members of the profession in the election of direct representatives.

The results of the election of direct representatives for England and Wales for the General Medical Council were given in our last issue, and were much as might have been anticipated. Dr. Henry Langley Browne, as Chairman of the Council of the British Medical Association, is well known to medical men all over the country, and was recognised by virtue of his experience and his concern in the general interests of the profession as a candidate eminently suited to represent them on the Council. The other two successful candidates—Dr. Latimer and Dr. McManus—were associated with Dr. Langley Browne in their election addresses and probably gained to some extent by distinction reflected from their more illustrious colleague. Dr. Latimer had the undivided support of Wales, and may be said to represent the strong desire for reform in the existing conditions of contract practice which especially obtain in mining and working-class districts. Dr. McManus, on the other hand, depended largely for support on the fact that he was a local candidate for the Metropolis. Mr. George Jackson, of Plymouth, and Mr. George Brown, of Callington, Cornwall, previous representatives on the Council, failed to be re-elected by quite a narrow margin of votes. The election figures are disappointing inasmuch as they do not display any marked increase of interest in the election by members of the profession. Dr. William Bruce, who has represented Scotland on the Medical Council for the past twenty years, has been superseded by Dr. Norman Walker, of Edinburgh. No doubt Dr. Norman Walker will discharge his duties faithfully, but it is a serious matter, in our view, that the profession in Scotland should have failed to recognise the extent and value of twenty years' service as a direct representative which Dr. William Bruce has so zealously rendered to his professional brethren. Dr. Bruce was one of the most valued and deservedly popular members of the Medical Council. He will be greatly missed on that body, and although there is sometimes a good deal to be said in favour of the principle of new blood, the enforcement of this principle cannot be welcomed where it entails the loss of great experience and capacity.

